# Thumbs Up! A Novel Use of the Acutrak Screw Fixation System for the Management of Triphalangeal Thumb

**Published:** 2010-03-29

**Authors:** Z. Ahmad, C. N. McGuiness

**Affiliations:** Odstock Centre for Burns and Plastic & Reconstructive Surgery, Salisbury District Hospital, Salisbury, Wiltshire SP2 8BJ, United Kingdom

## Abstract

**Objective:** The management of triphalangism provides the age-old problem of marrying maximal functional benefit with acceptable cosmesis. The objective was to discuss the rare abnormality of bilateral triphalangeal thumb, the surgical existing surgical approaches, and to present a case in a pediatric patient who had a good result with a relatively simple and straightforward method of surgically managing the deformity. **Method:** We present the case of a 16-year-old girl with isolated bilateral triphalangism of the thumbs. Her primary concern was the cosmetic deformity conferred by a delta middle phalanx, resulting in ulnar deviation of the thumb, in addition to the consequent reduced function of the thumb. Our approach to this problem was to perform a wedge osteotomy of the delta phalanx and a shave osteotomy of the articular surface of the distal phalanx, before using the Acutrak screw system to perform an arthrodesis. We present the first reported case in the literature. **Results:** This resulted in a near-anatomical correction of the deformity and consequent improvement of the cosmesis with a short anesthetic time, quick postoperative recovery, and very satisfied patient. **Conclusion:** We acknowledge that superior techniques exist; however, we also recognize that every case is unique and therefore, in this case the use of the Acutrak screw fixation system was the best option. It offers a noncomplex method of managing a complex condition. Short, safe, and effective surgery is always the best practice in managing pediatric patients.

A triphalangeal thumb is a relatively rare congenital abnormality with overall incidence reported as 1 in 25,000 live births, although it actually occurs more commonly than reported.^1^^-^3 Classically, two thirds of children exhibit the condition bilaterally, and usually there is a positive family history.^4^,^5^ There are however various types, one form of which is characterized by a short triangular or “delta” middle phalanx which often occurs sporadically and is typically unilateral.^5^

Treatment options vary from conservative management to excision and closing wedge osteotomy to partial and total resection to pollicization.^2^,^3^ Ultimately management is aimed primarily toward restoring basic hand function, power grasp, and precision pinch, and secondarily to improve cosmesis which can have behavioral and psychological implications.^3^,^5^

Interphalangeal arthrodeses can be performed in order to treat pain, joint instability, and congenital and acquired deformity.^6^,^8^ Compression and rigid fixation are thought to produce favorable outcomes with respect to fusion rates, time to union, and subsequent functionality.^6^,^7^ This is widely accepted as the gold standard for eliminating pain in arthritic joints.^6^,^7^ The merits of the various available techniques including Kirschner wire fixation can be argued on the basis of possible shorter operating times, less technically challenging operations, and acceptable fusion rates.^7^ On the contrary, however, there is considerable evidence in the literature stating that this method of fixation often leads to the complications of nonunion or delayed union.^6^,^7^ More recently, fixation using tension band wiring with Herbert and Acutrak (Acumed Inc, Beaverton, Oregon) screws has gathered favor because this technique has the advantage of offering more stability and higher rates of union when compared with other fixation techniques and is arguably technically as easy to perform.

## CASE REPORT

**Figure 2:**
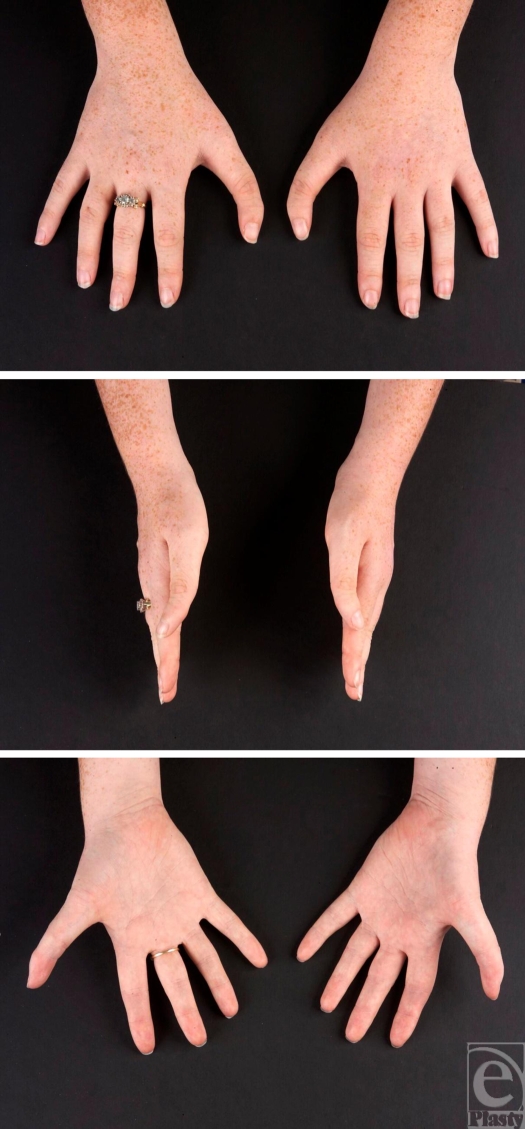
Preoperative photographs of both hands depicting the bilateral triphalangism and clinodactyly of the thumbs.

**Figure 3:**
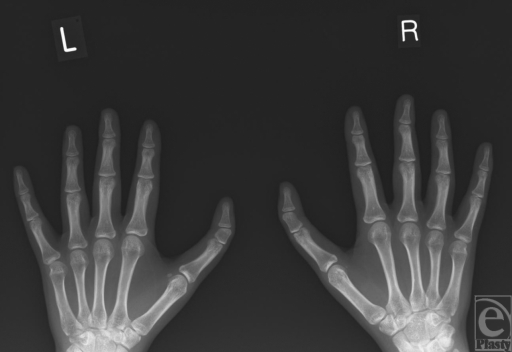
Preoperative radiograph of both hands showing the delta phalanx of the triphalangeal thumbs.

A right-handed, dominant 16-year-old girl presented to outpatients with a congenital deformity of both thumbs. She was otherwise fit and well. She had three sisters, all of whom were unaffected, and there was no family history of such abnormalities affecting the hand, limbs, cardiovascular, or gastrointestinal system. On examination, she had bilateral triphalangism, clinodactyly with ulnarward deviation in each thumb (Figs 2 and 3). Functionally, she complained of stiff distal interphalangeal joints (DIPJ) with a maximum of 10 degrees of passive flexion and extension, but had good active and passive flexion and extension at the proximal interphalangeal joints. The patient and the parental consent from the patient as well as the parents was sought and granted in the production of this article.

**Figure 4:**
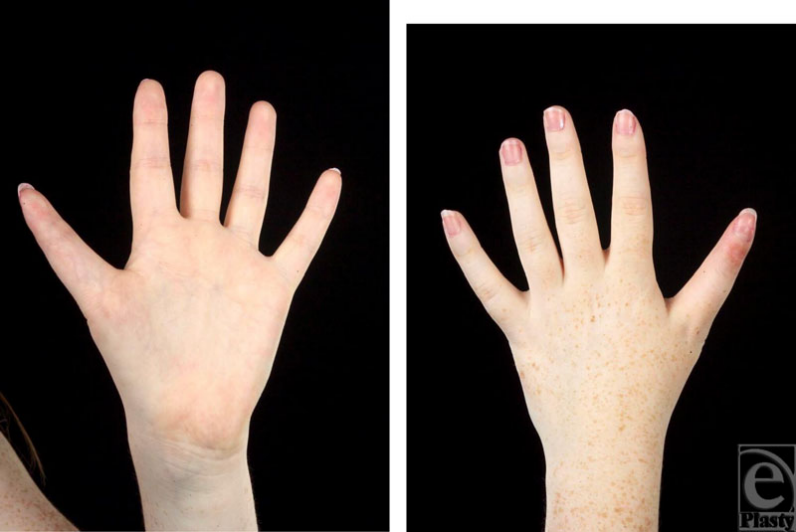
Postoperative photographs at 12 weeks.

**Figure 5:**
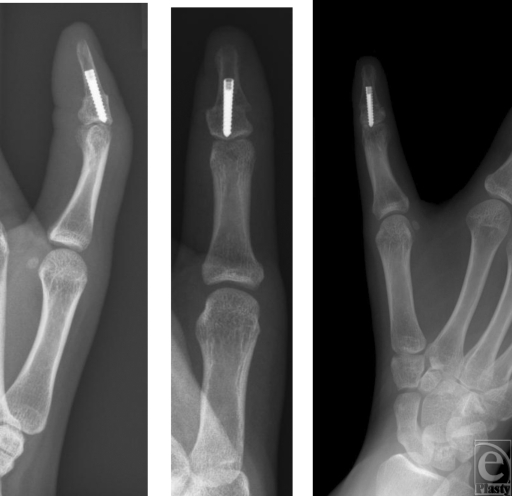
Postoperative radiographs at 12 weeks.

As the patient desired improved function and better cosmesis, she and her parents were keen on surgical correction. She subsequently underwent fusion of the DIPJ of the left thumb only initially, with Acutrak screw fixation system. A 14.0-mm titanium fusion screw device was used to fuse the joint across the articular surfaces of the joints fashioned postosteotomy. The DIPJ articular surface was excised via osteotomy, and the proximal end of the anomalous phalanx was then fused to the distal end of the distal phalanx to create a single phalanx (Figs 4 and 5). She made an uncomplicated recovery. Six weeks postcorrection, she had 60 degrees flexion at her left thumb interphalangeal joint and was delighted with the result, both with respect to her functional use and the appearance of her “normal” thumb. She opted for the correction of the contralateral deformity and is keen to pursue fashion at college.

## DISCUSSION

According to the Buck-Gramcko system, the treatment of type II triphalangism comprises excision of the short triangular bone, the delta fragment (Fig. 2) with a closing wedge osteotomy.^5^ Although this is largely the accepted method of management of type II anomalies, we feel the use of Acutrak screw fixation provides better results, in terms of surgical approach, namely quicker operation times, shorter pediatric anesthesia, better intraoperative visualization, and perhaps most importantly, a one-stage procedure. The senior author (C.N.McG.) feels that the Acutrak screw maintains better compression through cyclic loading compared to Herbert or AO screws, (AO Foundation, Switzerland), and as a result, higher rates of union are achieved. Further, due to the technical ease of the insertion procedure, as well as the Acutrak screw fixation system, the procedure can be performed quickly and safely giving good results. Similarly, with respect to postoperative outcome, better joint fusion and alignment, subsequent stability, function, and cosmesis, together with patient satisfaction, are achievable with this technique, which makes it arguably superior to existing methods of management.

## Figures and Tables

**Figure 1:**
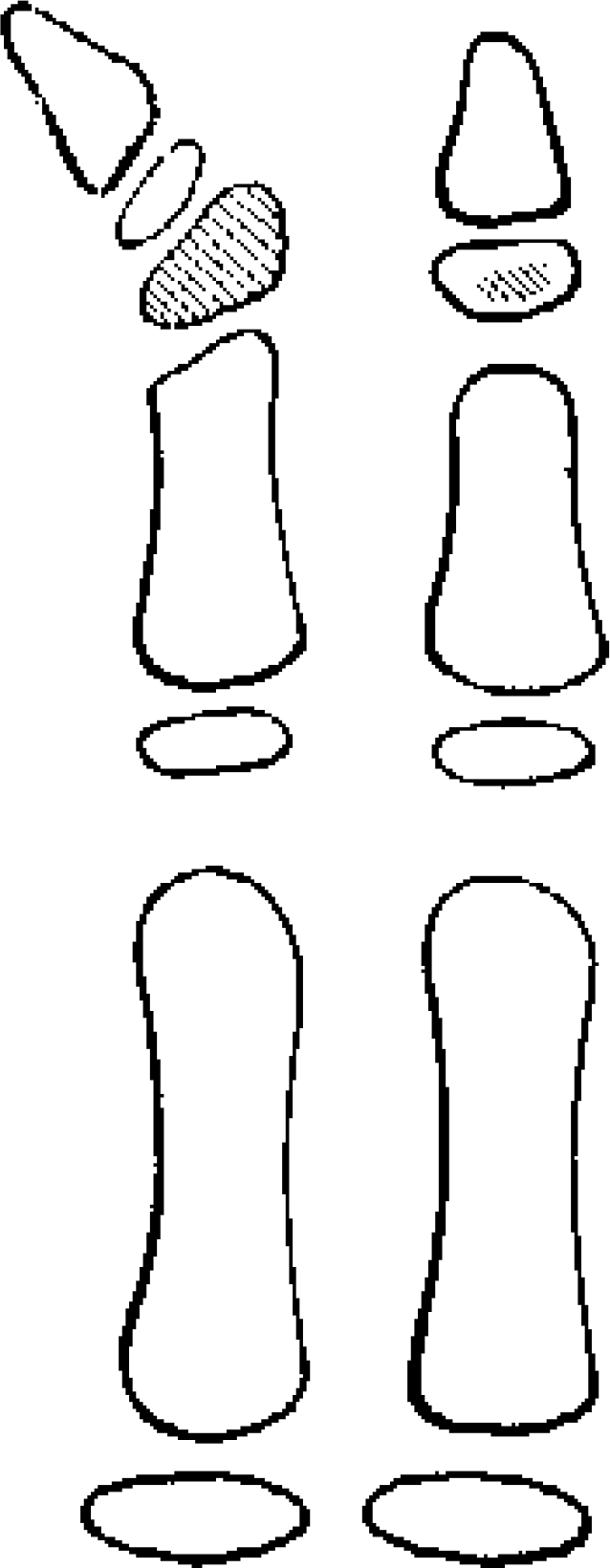
This illustration shows the shaded rudimentary bone developing as an asymmetric secondary ossification center that is larger than normal. Triphalangeal thumb. From Gupta et al.^9^.
